# Development of Mitomycin C-Loaded Nanoparticles Prepared Using the Micellar Assembly Driven by the Combined Effect of Hydrogen Bonding and π–π Stacking and Its Therapeutic Application in Bladder Cancer

**DOI:** 10.3390/pharmaceutics13111776

**Published:** 2021-10-25

**Authors:** Lingling Qi, Chao Liu, Yingying Zhang, Zheao Zhang, Hongxia Duan, Heming Zhao, Xin Xin, Liqing Chen, Mingji Jin, Youyan Guan, Zhonggao Gao, Wei Huang

**Affiliations:** 1 State Key Laboratory of Bioactive Substance and Function of Natural Medicines, Department of Pharmaceutics, Institute of Materia Medica, Chinese Academy of Medical Sciences and Peking Union Medical College, Beijing 100050, China; qilingling@imm.ac.cn (L.Q.); chaoliu@imm.ac.cn (C.L.); zyy@imm.ac.cn (Y.Z.); zhangzheao@imm.ac.cn (Z.Z.); dhx@imm.ac.cn (H.D.); zhaoheming@imm.ac.cn (H.Z.); xinxin@imm.ac.cn (X.X.); chenliqing@imm.ac.cn (L.C.); jinmingji@imm.ac.cn (M.J.); 2 Department of Urology, National Cancer Center/National Clinical Research Center for Cancer/Cancer Hospital, Chinese Academy of Medical Sciences and Peking Union Medical College, Beijing 100021, China

**Keywords:** micelles, mitomycin C, bladder cancer, π–π stacking, hydrogen bonding interaction

## Abstract

Micelle is mainly used for drug delivery and is prepared from amphiphilic block copolymers. It can be formed into an obvious core-shell structure that can incorporate liposoluble drugs. However, micelles are not suitable for the encapsulation of water-soluble drugs, and it is also difficult to maintain stability in the systemic circulation. To solve these problems, a type of polymer material, Fmoc-Lys-PEG and Fmoc-Lys-PEG-RGD, was designed and synthesized. These copolymers could self-assemble into micelles driven by π–π stacking and the hydrophobic interaction of 9-fluorenylmethoxycarbony (Fmoc) and, at the same time, form a framework for a hydrogen-bonding environment in the core. Mitomycin C (MMC), as a water-soluble drug, can be encapsulated into micelles by hydrogen-bonding interactions. The interaction force between MMC and the polymers was analyzed by molecular docking simulation and Fourier transform infrared (FTIR). It was concluded that the optimal binding conformation can be obtained, and that the main force between the MMC and polymers is hydrogen bonding. Different types of MMC nanoparticles (NPs) were prepared and the physicochemical properties of them were systematically evaluated. The pharmacodynamics of the MMC NPs in vitro and in vivo were also studied. The results show that MMC NPs had a high uptake efficiency, could promote cell apoptosis, and had a strong inhibitory effect on cell proliferation. More importantly, the as-prepared NPs could effectively induce tumor cell apoptosis and inhibit tumor growth and metastasis in vivo.

## 1. Introduction

Bladder cancer is the most common malignant tumor of the urinary system, and it is also one of the ten most commonly occurring tumors in the world [1,2]. Approximately 90% of bladder cancer is superficial growth, having the characteristics of polycentricity, easy recurrence, and metastasis and, unfortunately, the five-year survival rate of patients with stage Ⅳ bladder cancer is only 15% [3]. Presently, the clinical treatment of bladder cancer is mainly surgery, supplemented by radiotherapy and chemotherapy, but there is no ideal targeted therapy [4,5].

Mitomycin C (MMC) has been used for the prevention and treatment of the postoperative recurrence of non-muscle-invasive bladder cancer since 1996 [6]. So far, MMC is still one of the main chemotherapeutic agents by perfusion used in the prevention of the postoperative recurrence of bladder cancer [7]. Infusion chemotherapy injects cytotoxic drugs into the bladder through bladder instillation, and then directly kills tumor cells, thereby reducing the risk of tumor recurrence and progression. However, MMC has systemic toxicity, and myelosuppression is one of the serious adverse reactions. Its toxicity to the heart, lungs, and other organs has also been reported [8]. These toxic side effects greatly limit the application of MMC.

The delivery of MMC through nanodrug delivery systems (including nanoparticles, polymer micelles, liposomes, and dendrimers, etc.) can significantly improve its targeting, change the biological distribution of the drugs, reduce the distribution of the drugs in normal tissues, and also increase the accumulation and retention time of drugs in tumor sites [9,10,11,12,13]. Because of the water-soluble characteristics of MMC, most MMC is first modified into prodrugs or lipid complexes, and then loaded with liposomes or encapsulated with nanoparticles (NPs) by the double emulsion solvent diffusion technique, or others [13,14,15,16,17]. However, most of the drug delivery methods of MMC reported in the literature have a low encapsulation efficiency, and it is difficult to maintain the stability of the drug in the carrier [16,17]. Moreover, modifying MMC as a prodrug not only increases the preparation difficulty, but also may reduce the pharmacological activity of MMC.

**Scheme 1:**
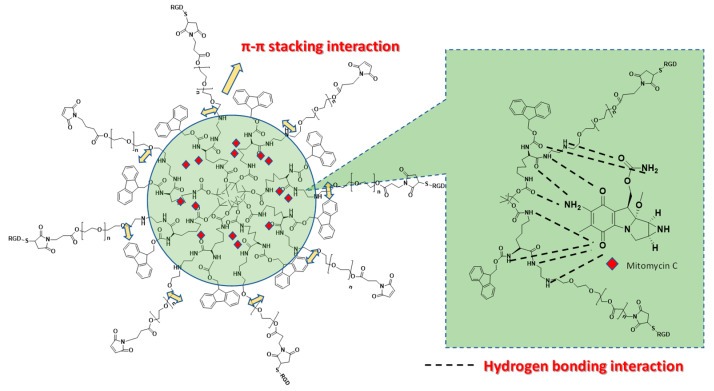
Schematic diagram of MMC-loaded micellar assembly driven by the combined effect of hydrogen bonding and π–π stacking.

Therefore, we designed and synthesized a type of polymer material, Fmoc-Lys-PEG and Fmoc-Lys-PEG-RGD. These copolymers can self-assemble into micelles driven by π–π stacking and the hydrophobic interaction of the Fmoc motif and, at the same time, form the framework of a hydrogen-bonding environment in the core, as shown in Scheme 1. Water-soluble MMC could be encapsulated inside the carrier by the hydrogen-bonding force, and we also confirmed it by means of molecular simulation. Given that RGD peptides can target integrin receptor that is highly expressed on the endothelial cells of neocapillaries in malignant tumors [18,19], according to the defects of MMC treatment in bladder cancers, c(RGDfc) peptides were also introduced on the surface of the carrier [20], thus further improving the therapeutic effect on bladder cancer. It is hoped that, through our work, we can provide some ideas for the micellar encapsulation of hydrophilic drugs, and also provide some reference for the treatment of bladder cancer.

## 2. Materials and Methods

### 2.1. Materials

Fmoc-Lys (Boc)-OH (99%), HEPEs, CDCl_3_, and Indole-3-acetic acid (IAA) were procured from Sigma-Aldrich (St. Louis, MO, USA). MAL-PEG2000-NH_2_ (98%) was obtained from tansh-tech Ltd., (Guangzhou, China). C (RGDfc) was procured from Tianjin Nuokang Xinda Technology Ltd., (Tianjin, China). Mitomycin C (MMC) was bought from Shanghai Shifeng Biotechnology Co., Ltd., (Shanghai, China). Cell Counting Kit-8 (CCK-8) was bought from DOJINDO Molecular Technologies, Inc. (Shanghai, China). DMEM, 0.25% trypsin, and PBS Buffer were bought from Gibco, (Waltham, MA, USA); FBS was purchased from Beijing Xigong Biotechnology Co., Ltd. (Beijing, China). An Annexin V-FITC/PI Cell Apoptosis Kit was purchased from 4A Biotech Co., Ltd., (Beijing, China). All the other chemicals were of reagent grade without further purification.

MB49 cells (Hunan Fenghui Biotechnology Co., Ltd., Hunan, China) were cultured in DMEM medium containing 10% FBS and 5% CO_2_, at 37 °C. Female C57BL/6 mice (6–8 weeks old, SPF grade, 18–20 g) were purchased from the Beijing Charles River Laboratory Company. All experiments of animal-related study were reviewed and ethically approved by the Institute of Materia Medica, Chinese Academy of Medical Sciences (approval number: 00003368 (10 December 2020)).

### 2.2. Synthesis of Polymers

**Scheme 2:**
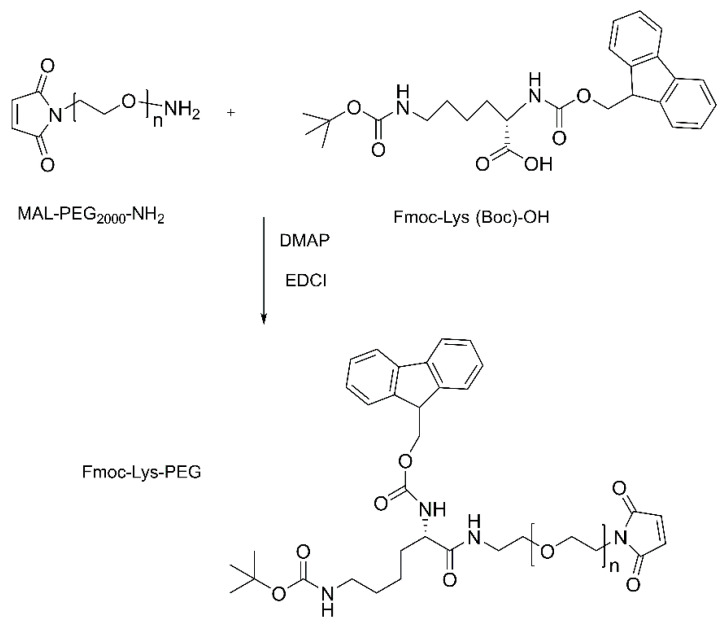
The synthetic route of Fmoc-Lys-PEG copolymer.

**Scheme 3:**
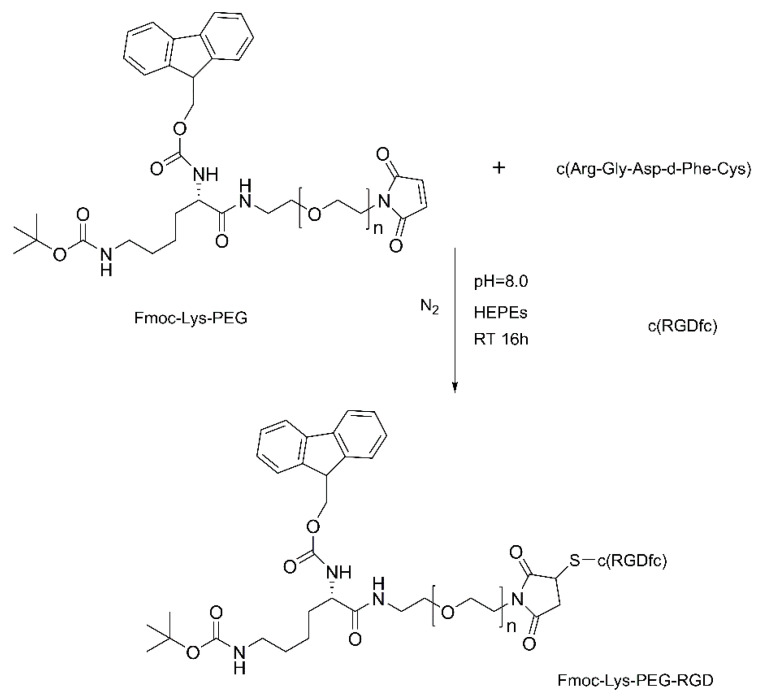
The synthetic route of Fmoc-Lys-PEG-RGD copolymer.

The synthetic routes of Fmoc-Lys-PEG and Fmoc-Lys-PEG-RGD are shown in Scheme 2 and Scheme 3. The Fmoc-Lys-PEG-Mal was synthesized by the esterification of Fmoc-Lys (Boc)-COOH and Mal-PEG-NH_2_ by the EDC/NHS coupling reaction. Firstly, Fmoc-Lys (Boc)-COOH (4 equiv.) and DMAP (0.2 equiv.) was dissolved with 5 mL anhydrous THF in a 25 mL round-bottomed flask under stirring. Then, EDCI (4 equiv.) was added under nitrogen atmosphere. After reaction for about 1 h, Mal-PEG-NH_2_ (1 equiv.) was added and the reaction was stirred for a further 24 h at room temperature under nitrogen atmosphere. After completion of the reaction, the reaction mixture was filtered and precipitated in about 50 mL cold methyl tertiary-butyl ether. The product was dried at 40 °C in vacuum until constant weight [21,22].

The Fmoc-Lys-PEG-RGD was synthesized by the Michael addition reaction of Fmoc-Lys-PEG and c(RGDfc). Firstly, Fmoc-Lys (Boc)-PEG (1 equiv.) and C (RGDfc) (1.5 equiv.), were dissolved with a HEPES-buffered saline (pH 8.0) solution in a 25 mL round-bottom flask. Then, the reaction was stirred at room temperature under the protection of nitrogen for 16 h. After the reaction, the reaction mixture was dialyzed in deionized water for 24 h, and then the white powder solid was obtained by freeze-drying [23].

### 2.3. Characterization of Polymers

The polymer structure was analyzed by an ^1^H NMR spectra (400 MHz) spectrometer instrument (Varian Inc., Troisdorf, Germany) with CDCl_3_ as the solvent. 10 mg Fmoc-Lys-PEG and Fmoc-Lys-PEG-RGD were dissolved in 600 µL CDCl_3_, and their molecular structures were confirmed by nuclear magnetic resonance (^1^H-NMR). Matrix-assisted laser desorption/ionization time-of-flight mass-spectrometry (MAL-DI-TOF-MS) was conducted to determine the molecular weight distribution of the polymers, in which 3- indoleacetic acid (IAA) was used as the matrix.

### 2.4. Preparation of NPs

MMC nanoparticles (NPs) can be prepared by the thin-film hydration method. Briefly, Fmoc-Lys-PEG and Fmoc-Lys-PEG-RGD, with different molar ratios (9:1 and 10:0, respectively), were placed in a round-bottomed flask, dissolved with a certain amount of MMC methanol solution, and then stirred for 30 min. After solvent evaporation, different hydration media were added, then MMC-loaded non-RGD nanoparticles (MMC NPs), and MMC-loaded RGD nanoparticles (MMC RGD-NPs), were obtained after incubation for about 30 min.

### 2.5. Characterization of NPs

The particle size and zeta potential of the NPs were measured by dynamic light scattering and electrophoretic light scattering (Zeta-sizer Nano ZS90; Malvern Instruments Ltd., Malvern, UK), and the morphology of the MMC RGD-NPs was observed by transmission electron microscope (Hitachi Hmur7650, Hitachi Company, Tokyo, Japan).

The entrapment efficiency (EE%) and drug loading (DL%) of MMC in the MMC NPs and the MMC RGD-NPs were determined by the ultrafiltration centrifugation method. Briefly, 0.5 mL of the MMC NPs or MMC RGD-NPs were placed in an ultrafiltration centrifuge tube (Millipore, M = 10,000 Da), centrifuged for 20 min under the condition of 1485 g, and the filtrate was analyzed by HPLC (Agilent 1260, Agilent Technologies, Santa Clara, CA, USA) with a reverse-phase 18 (RP−18) column (Hibar^®®^ 250 × 4.6 mm, 5 µm, Merck KGaA, Darmstadt, Germany). The column temperature was 35 °C, eluted with mobile phase (acetonitrile/water, 25/75, v/v), at a flow rate of 1 mL/min, and the column effluent was detected using an ultraviolet detector at λ _max_ of 365 nm.

(1) $$\mathrm{EE}\%=\frac{\mathrm{Weight}\;\mathrm{of}\;\mathrm{encapsulated}\;\mathrm{drug}}{\mathrm{Weight}\;\mathrm{of}\;\mathrm{total}\;\mathrm{drug}}\times 100\%$$

(2) $$\mathrm{DL}\%=\frac{\mathrm{Weight}\;\mathrm{of}\;\mathrm{encapsulated}\;\mathrm{drug}}{\mathrm{Weight}\;\mathrm{of}\;\mathrm{total}\;\mathrm{drug}\;\mathrm{and}\;\mathrm{vector}}\times 100\%$$

The infrared spectra of the MMC, the blank NPs, and the MMC NPs were recorded on a Nicolet 5700 Fourier transform infrared (FTIR) spectrometer (Thermo Fisher Scientific, Waltham, MA, USA) by the KBr pellet technique.

### 2.6. Molecular Docking Simulation of Polymers and MMC

Molecular docking software, AutoDock Vina (Version 1.5.6 Molecular Graphics laboratory, La Jolla, CA, USA) [24,25], was used to simulate the docking between MMC and Fmoc-Lys-PEG. Firstly, the molecular 3D structure file of MMC was retrieved and downloaded from PubChem, and the file format was converted to “.pdb” format by Open BableGUI2.2.1. The 3D structure of Fmoc-Lys-PEG was drawn by ChemBiodraw Ultra and ChemBio3D Ultra software, and the file format was also transformed into “.pdb” format. By setting up three different docking modes, the optimal binding conformation, binding energy, and interaction force between material and drug were analyzed by AutoDock Vina: (1) Fmoc-Lys-PEG was the ligand and MMC was the receptor; (2) Fmoc-Lys-PEG was the ligand and MMC was the receptor (Fmoc limited binding pocket); (3) MMC was the ligand and Fmoc-Lys-PEG was the receptor.

### 2.7. The Release Behavior of NPs

The in vitro release of MMC NPs and MMC RGD-NPs was determined by the dialysis diffusion method. Firstly, MMC solution and MMC NPs, and the MMC RGD-NP suspension solutions, with an MMC concentration of 1 mg/mL, were prepared. A 0.5 mL sample solution was accurately measured and transferred to a dialysis bag (MWCO10 KD), then placed in a centrifuge tube containing 10 mL PBS buffer (containing 0.5% Tween−80) and oscillated at a constant temperature of 37 °C and 100 rpm. An amount of 0.5 mL of the release medium was taken out regularly, and 0.5 mL of fresh release medium was added at the same time. The release medium was centrifuged at 13,362× *g* for 10 min, and the concentration of MMC was determined by HPLC [12].

### 2.8. In Vitro Hemolysis Testing

Blood was taken from the orbit of C57BL/6 mice and stirred with bamboo sticks to remove fibrin from the blood. Then the blood was washed by centrifugation with 0.9% normal saline at 371 g for 10 min. This process was repeated several times until the supernatant no longer showed red. The precipitate was diluted with 0.9% saline into a 0.2% (v/v) red blood cell suspension.

Sample solutions with MMC concentrations of 0.10, 0.25, 0.5, 0.75, and 1.0 mg/mL were prepared with 0.9% normal saline, and an equal volume of red blood cell suspension was added. Deionized water was set as the positive control group, and 0.9% saline was set as the negative control group. The mixture was evenly mixed and incubated in an air gas bath oscillator at 37 °C for 1 h, then centrifugated at 371× *g* for 10 min. The absorbance (A) of the supernatant was measured at λ = 575 nm, and the hemolysis of each group was calculated.

### 2.9. Cell Viability Assay of NPs

The cytotoxicity of NPs was investigated using MB49 cells by a tetrazolium-8-[2-(2-methoxy-4-nitrophenyl)-3-(4-nitrophenyl)-5-(2,4-disulfophenyl)-2H-tetrazolium] monosodium salt (CCK-8) assay. MB49 cells were incubated in 96-well plates, at a density of 5000 cells per well, for 24 h, and different concentrations of blank solution were added to continue to culture for 24 h or 48 h. Then, CCK-8 was added for further incubation for 2 h, and an OD value at λ = 450 nm was measured by microplate reader (BioTek Instruments, Winooski, VT, USA).

### 2.10. Proliferation Inhibition Effect of NPs

The CCK-8 assay was also used to evaluate the inhibitory capacity of MMC RGD-NPs against cell proliferation, after incubation for 24 and 48 h, compared with MMC solution. MB49 cells were incubated in 96-well plates at a density of 5000 cells per well for 24 h, and MMC solution and MMC RGD-NPs with different concentrations of MMC were added for 24 h or 48 h. Then, CCK-8 was added and incubation continued for 2 h. The OD value at λ = 450 nm was determined by microplate reader (BioTek Instruments, Winooski, VT, USA).

(3) $$\mathrm{Cell}\;\mathrm{viability}\%=\frac{{\mathrm{OD}}_{\mathrm{test}}-{\mathrm{OD}}_{\mathrm{blank}}}{{\mathrm{OD}}_{\mathrm{control}}-{\mathrm{OD}}_{\mathrm{blank}}}\times 100\%$$

### 2.11. Cellular Uptake Studies

C6-labeling NPs, or RGD-NPs, were prepared by the thin-film hydration method. Firstly, C6 was dissolved in dichloromethane (CH_2_Cl_2_) with a concentration of 1 mg/mL. Then, Fmoc-Lys-PEG and Fmoc-Lys-PEG-RGD, with different molar ratios (9:1 and 10:0, respectively), were placed in a round-bottomed flask, dissolved with a certain amount of C6 CH_2_Cl_2_ solution, and stirred for 30 min. After solvent evaporation, different hydration media was added, and C6-loaded non-RGD nanoparticles (C6 NPs), and C6-loaded RGD nanoparticles (C6 RGD-NPs), were obtained after incubation for about 30 min. The C6-labeling NPs were diluted to a certain concentration in serum-free medium and then used for cell uptake evaluation.

Cellular uptake was qualitatively investigated for the MB49 cells by confocal laser scanning microscopy. Firstly, the MB49 cells were seeded into 12-well plates at a density of 15 × 10^4^ cells/mL and cultured at 37 °C and 5% CO_2_ for 24 h. Then, C6 solution, C6 NPs, and C6 RGD-NPs with a C6 concentration of 1µg/ mL were added. After a 2 h coculture, the cells were washed with cold PBS three times and fixed with 4% paraformaldehyde for 10 min. The cells were washed with cold PBS again three times and continually stained with 1 μg/mL of DAPI for 15 min. The uptake of cells was finally observed under a laser confocal microscope (TCSSP2, Leica, Wetzlar, Germany). The time-dependence of the cell uptake, and the uptake of C6 NPS at different incubation times, such as 15, 60, and 120 min, was also observed by laser confocal microscope (TCS SP2, Leica).

The intracellular uptake efficiency of C6-labeled NPs was quantitatively determined by flow cytometry. MB49 cells were seeded into 12-well plates at a cell density of 15 × 10^4^ cells/mL and incubated at 37 °C and 5% CO_2_ for 24 h. After coculture for 120 min, the cells were washed with cold PBS three times. The cells were collected by trypsin digestion cells and resuscitated with PBS. The uptake of coumarin 6 NPs was investigated by FACS Calibur flow cytometry (Becton Dickinson, Franklin Lake, NJ, USA). The effects of different incubation times (15, 60, and 120 min) on the uptake of C6-NPs were also investigated by flow cytometry to explore the time dependence of C6-NP uptake.

### 2.12. Cell Apoptosis Assay

The apoptosis of MB49 cells caused by MMC RGD-NPs was quantitatively analyzed by the Annexin V-FITC/ PI double staining assay. Firstly, MB49 cells were inoculated in 12-well plates at a density of 12 × 10^4^ cells/mL for 24 h. Then, MMC solution, MMC NPs, and MMC RGD-NPs with an MMC concentration of 1 µg/mL were added to culture for 24 h. The cells were washed with cold PBS three times, and then digested and collected. After dispersion in binding buffer, the cells were treated with Annexin V-FITC, according to the protocols of Annexin V-FITC and the PI staining manufacturer. The samples were analyzed by the FACS Calibur flow cytometer (Becton Dickinson, Franklin Lake, NJ, USA).

### 2.13. Antitumor Evaluation of NPs In Vivo

MB49 cells in the logarithmic growth stage were taken and digested by 0.25% trypsinase, and cell precipitation was obtained by centrifugation. The cells were resuspended with PBS and the cell concentration was controlled at 5 × 10^6^ cells/mL and stored on ice. Female C57BL/6 mice were subcutaneously injected with 100 μL cell suspensions under the right armpit forelimb, and five unseeded C57BL/6 mice were selected as the blank group. The tumor size of the mice was measured every other day with a vernier caliper until the tumor volume reached about 100 mm^3^. The tumor-bearing mice were randomly grouped into four groups (*n* = 5), as follows: saline, MMC solution, MMC NPs, and MMC RGD-NPs. The tumor-bearing mice were administered through the tail vein seven times, every three days. The volume of each injection was 200 µL and the dose of MMC was set as 3.0 mg/kg. The changes in tumor volume and the body weight of the mice were monitored at the same time, during administration. All tumor-bearing mice were killed three days after the seventh administration by cervical dislocation. The tumors in each group were isolated, weighed, and recorded, and the tumor inhibition rate was calculated. The tumor was fixed with neutral formaldehyde for 24 h and photographed in group. The tumors in each group were stained with TUNEL after the paraffin section to detect the apoptosis of tumor cells. The tissues (heart, liver, spleen, lung, kidney) were also isolated and treated with 4% formaldehyde tissue fixative and stained with H&E.

### 2.14. Statistical Analysis

All the data in this article were presented as “mean ± SD”. SPSS Statistics 19 (version 4.0.100.1124; SPSS Inc., IBM Company, Armonk, NY, USA) was used for data analysis. The Student’s *t*-test was used to evaluate the statistical differences between the two groups, as follows: * *p* < 0.05, ** *p* < 0.01, and *** *p* < 0.001 represent the degree of significant difference.

## 3. Results

### 3.1. Characterization of Polymers

**Figure 1:**
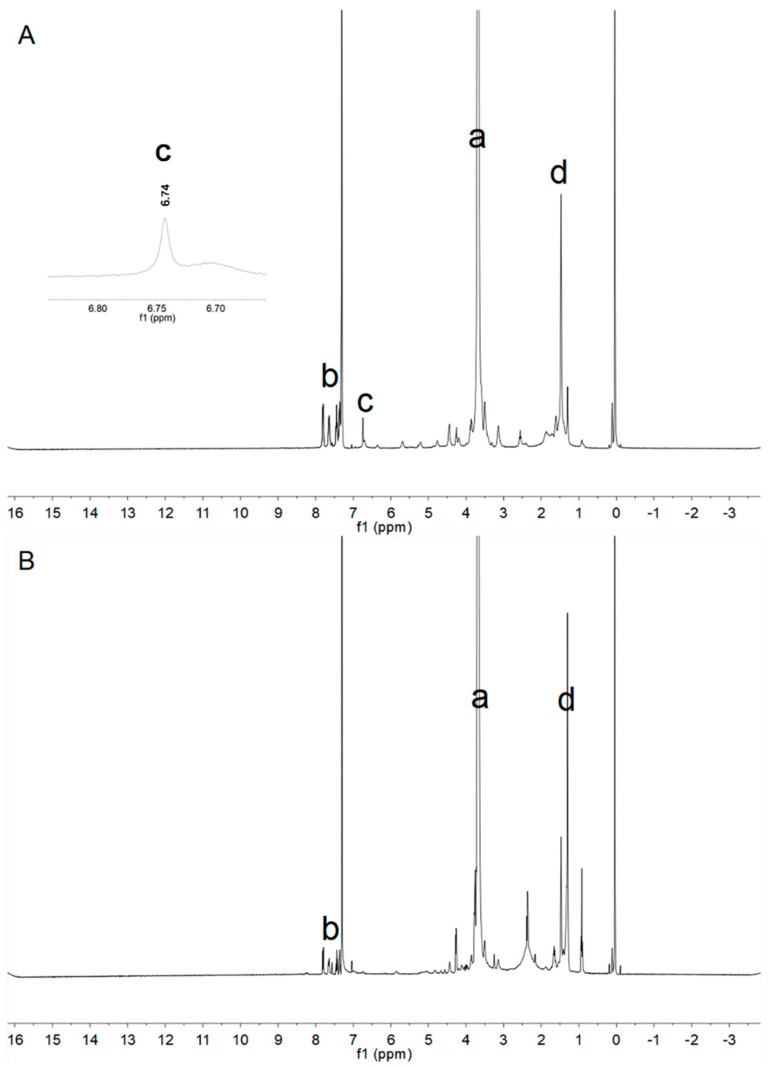
Determination results of Fmoc-Lys-PEG and Fmoc-Lys-PEG-RGD. (**A**) ^1^H-NMR spectra of Fmoc-Lys-PEG. (**B**) ^1^H-NMR spectra of Fmoc-Lys-PEG-RGD. a: 3.5–3.8 ppm, PEG; b: 7.0–7.3 ppm, Fmoc; c: 6.7 ppm, Mal; d: 1–2 ppm, Boc.

To identify the chemical structure of this copolymer, ^1^H NMR spectroscopy and MALDI-TOF-MS were conducted. As shown in the ^1^H-NMR spectra of Fmoc-Lys-PEG in Figure 1A, the signals at 3.5–3.8 ppm were attributed to protons of the methylene in PEG, while the signals of 6.7 ppm were attributed to the protons of the terminal Maleimide (Mal) of PEG, and the signals of the benzene ring protons of Fmoc were at 7.0−7.3 ppm. As shown in Figure 1B, the signals of 6.7 ppm in the ^1^H-NMR spectra disappeared, which indicates that Mal was successfully reacted with RGD.

**Figure 2:**
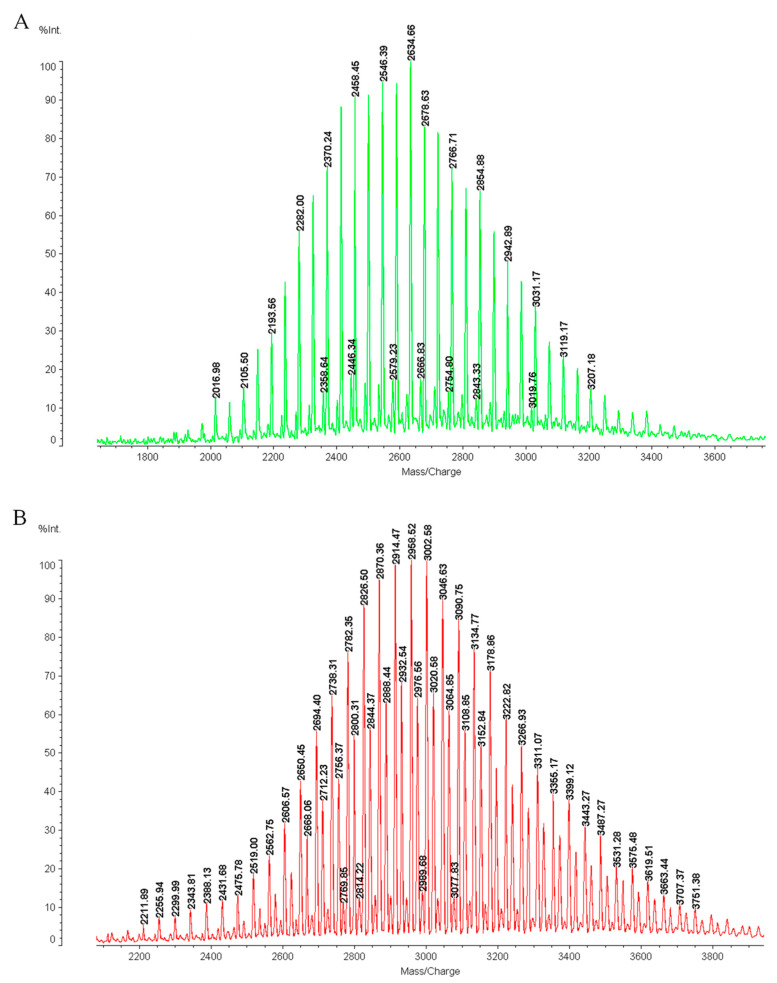
Determination results of Fmoc-Lys-PEG and Fmoc-Lys-PEG-RGD. (**A**) MALDI-TOF-MS spectra of Fmoc-Lys-PEG. (**B**) MALDI-TOF-MS spectra of Fmoc-Lys-PEG-RGD.

The MALDI-TOF-MS results of Fmoc-Lys-PEG are shown in Figure 2A. The detection peak value of Fmoc-Lys-PEG is mainly concentrated around 2634.7 Da, with high purity and no macromolecular impurities, which is close to the theoretical value. As shown in Figure 2B, the detection peak of Fmoc-Lys-PEG-RGD, mainly concentrated around 2955.8 Da, was also close to the theoretical molecular weight. Through the comparison and verification of ^1^H NMR data, it can be confirmed that the two materials were synthesized successfully.

### 3.2. Characterization of NPs

When the concentration of MMC was 1 mg/mL, and the molar ratio of the drug and carrier was 1:2.5, and with the particle size and the polydispersity index (PDI) as indicators, the hydration medium was screened, and the results are shown in Table 1. As can be seen from the table, when PBS was used as the hydration medium, the particle size and PDI data of the prepared NPs were better, and PBS was finally selected as the hydration medium.

The particle size and the PDI were used as indicators to screen the molar ratios of the drugs and carriers, and the results are shown in Table 2. As can be seen from the table, the particle size gradually increased with the increase of drug loading. When the molar ratio of the drug and carrier is 2.5:1, the particle size and PDI of the prepared NPs are smaller, so 2.5:1 was selected as the molar ratio of the drug and carrier.

MMC nanoparticles can be prepared by the thin-film hydration method. As shown in Figures S1A and S2A, the size of the MMC NPs was 115.5 ± 6.4 nm, with a PDI of 0.219 ± 0.027, and a zeta-potential of −1.17 ± 0.04 mV. As shown in Figures S1A and S2A, the size of the MMC RGD-NPs was 107.6 ± 2.2 nm, with a PDI of 0.137 ± 0.013, and a zeta-potential of −0.59 ± 0.07 mV.

**Figure 3:**
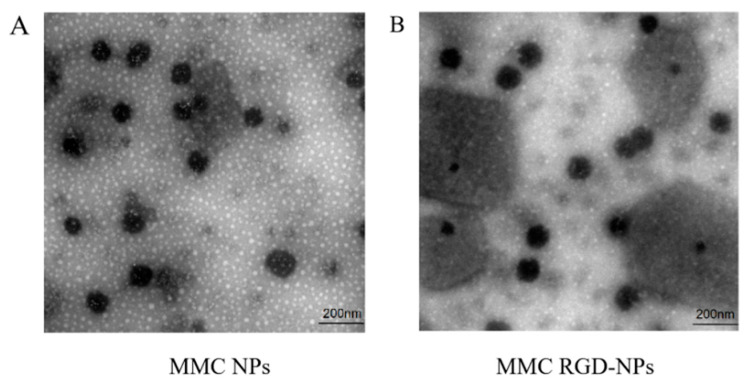
TEM image of MMC NPs (**A**) and MMC RGD-NPs (**B**).

Transmission electron microscopy (TEM) was also employed to investigate the micro-nano structures of these micelles, as shown in Figure 3. The MMC NPs and MMC RGD-NPs exhibit a spherical morphology and the particles are relatively regular and uniform in size.

**Figure 4:**
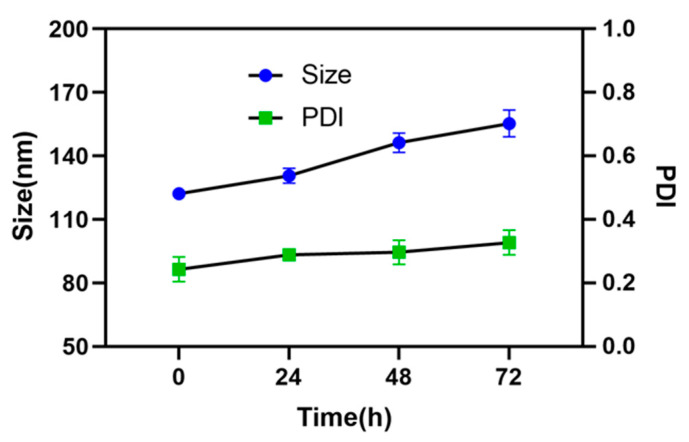
The result of stability of MMC NPs in 4 °C within 72 h.

**Figure 5:**
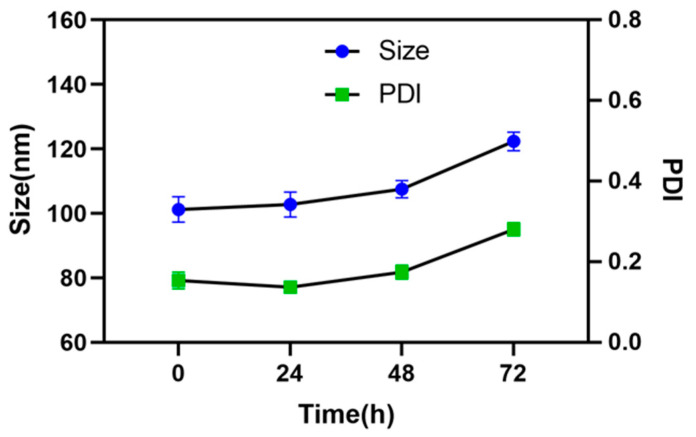
The result of stability of MMC RGD-NPs in 4 °C within 72 h.

The stability of micelles is of great significance to the application of micelles. As can be seen from Figure 4 and Figure 5, the particle size and PDI of NPs can remain basically stable within 48 h under the condition of 4 °C. However, with the extension of time, the particle size and PDI of the carrier will gradually become larger.

The entrapment efficiency (EE%) and drug loading (DL%) of the NPs were evaluated by the ultrafiltration centrifugation method, and the drug content was determined by high-performance liquid chromatography (HPLC). The EE% of MMC RGD-NPs, and MMC NPs, was (89.90 ± 0.18) % and (89.55 ± 0.15) %, respectively, and the DL% was (4.29 ± 0.10) % and (4.32 ± 0.10) %, respectively.

In order to demonstrate the formation of hydrogen bonding between MMC and Fmoc-Lys-PEG, FTIR analysis of the MMC, the FACS Calibur flow cytometer, and MMC NPs were also carried out. As shown in Figure S3, the peaks of MMC are mostly coincident with Fmoc-Lys-PEG, and the amides peak of the NPs was 3429.3 cm^−1^ (N-H), 1711.1 cm^−1^ (amide I), 1548.3 cm^−1^ (amide II), and 1250.9 cm^−1^ (amide III), while the amides peak of the MMC-NPs was shifted to 3421.3 cm^−1^ (N-H), 1711.1 cm^−1^ (amide I), 1529.4 cm^−1^ (amide II), and 1243.2 cm^−1^ (amide III).

### 3.3. In Vitro Release of NPs

**Figure 6:**
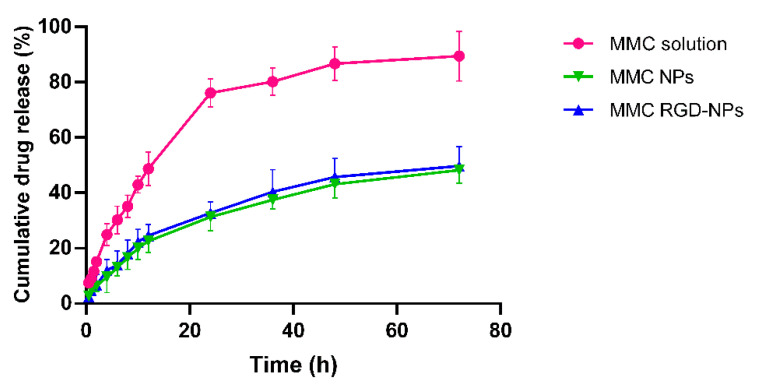
Study of release behavior of MMC RGD-NPs, in pH 7.4, in vitro.

Since MMC is unstable under acid-base conditions [26,27,28], we only studied the in vitro release behavior of MMC NPs and MMC RGD-NPs, in phosphate buffer with pH = 7.4, by simulating the normal physiological environment of the human body. As shown in Figure 6, both MMC NPs and MMC RGD-NPs were slow in drug release with similar release curves, and the cumulative release rate of the MMC NPs and the MMC RGD-NPs within 72 h was less than 50%, while that of the MMC solution was more than 80%. It can be seen that these prepared NPs have sustained release properties and can be used for the delivery of antitumor drugs.

### 3.4. Molecular Docking Simulation Analysis

**Figure 7:**
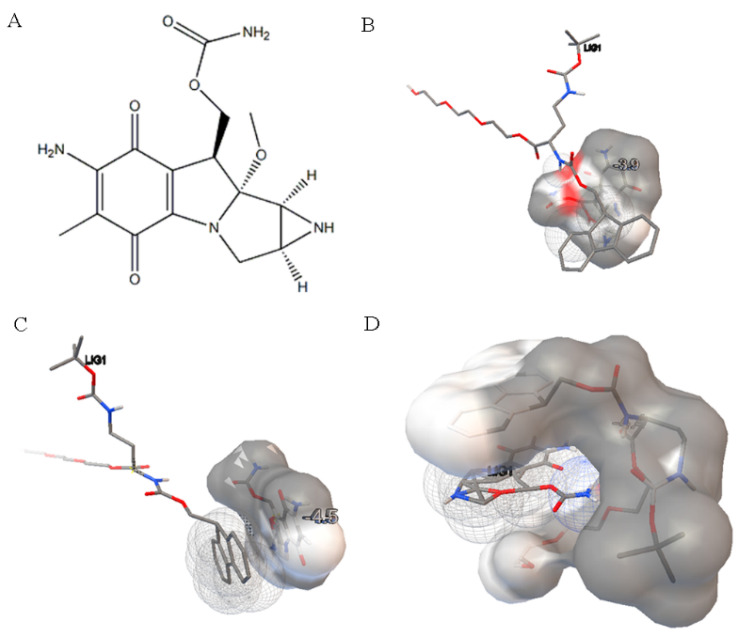
Prediction and analysis of the MMC-loading mechanism of nanoparticles. (**A**) The structure of MMC. (**B**) Fmoc-Lys-PEG was the ligand and MMC was the receptor. (**C**) Fmoc-Lys-PEG was the ligand and MMC was the receptor (Fmoc limited binding pocket). (**D**) MMC was the ligand and Fmoc-Lys-PEG was the receptor.

The molecular docking software, AutoDock Vina, was used to simulate the docking between MMC and the polymers. The optimal binding conformation and binding energy are given for each docking method are shown in Figure 7. We can judge the affinity between two molecules according to the binding energy. The lower the binding energy, the greater the affinity and the more stable the conformation. When Fmoc-Lys-PEG was the ligand and MMC was the receptor (Figure 7B), the lowest binding energy was −3.9 Kcal/mol. When we limited the Fmoc as a single binding pocket (Figure 7C), the lowest binding energy was −4.5 Kcal/mol. When MMC was the ligand and Fmoc-Lys-PEG was the receptor (Figure 7D), the lowest binding energy was −4.4 Kcal/mol. The optimal binding conformations of the three docking modes are shown in Figure 7B–D. The molecular docking interaction force between MMC and Fmoc-Lys-PEG was also detected by AutoDock Vina, and it was found that the interaction force that existed between them was mainly hydrogen bonding.

### 3.5. Hemocompatibility of NPs

In vitro hemolysis testing was employed to evaluate the biocompatibility of NPs. As shown in Table 3, the hemolysis degree of MMC RGD-NPs was less than 5% when the MMC concentration was 1.0 mg/mL, indicating good biocompatibility of the materials.

### 3.6. Biocompatibility of NPs

**Figure 8:**
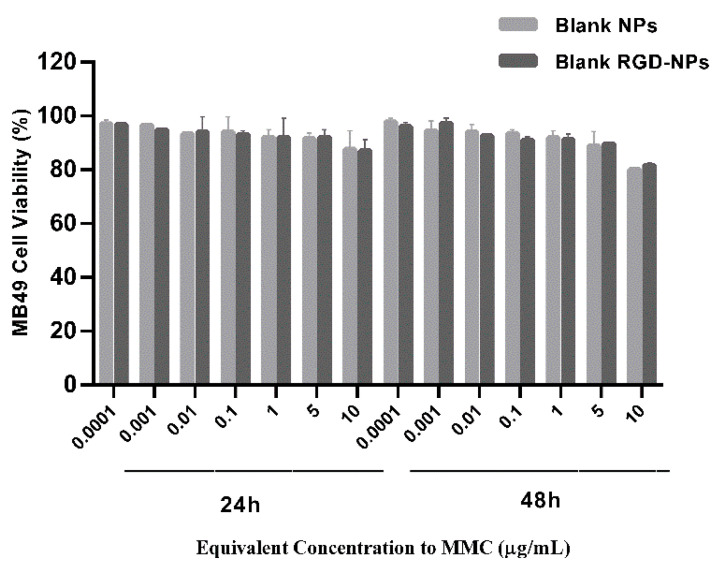
Cytotoxicity of blank NPs and blank RGD NPs after incubation for 24 and 48 h against MB49 cells (*n* = 6).

The cytotoxicity of NPs was also investigated using MB49 cells by a tetrazolium-8-[2-(2-methoxy-4-nitrophenyl)-3-(4-nitrophenyl)-5-(2,4-disulfophenyl)-2H-tetrazolium] monosodium salt (CCK-8) assay. As shown in Figure 8, when the equivalent concentration of MMC was 0.0001~10 μg/ mL, the relative concentration of the blank micelle was nontoxic compared with the control group, and the cell activity was maintained above 80%, indicating that the micelle had good biocompatibility and was suitable for drug delivery application.

### 3.7. Proliferation Inhibition Effect of NPs

**Figure 9:**
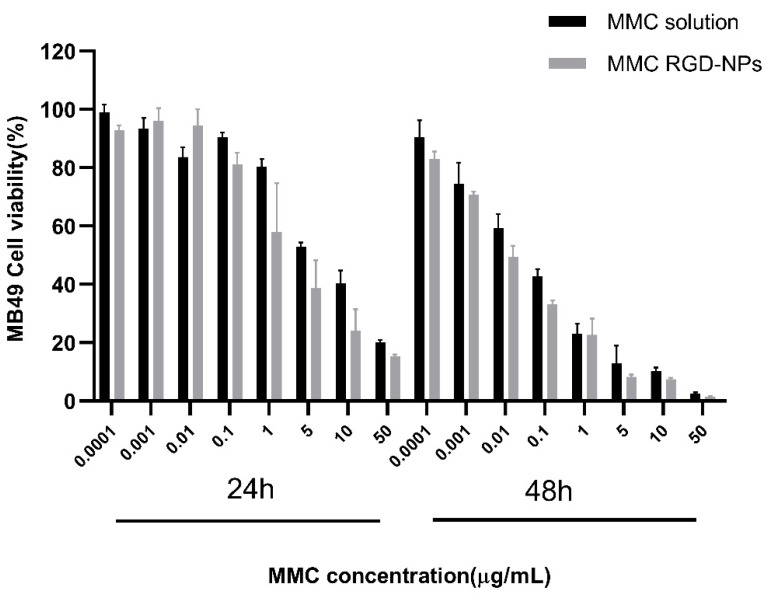
The cell viability of MB49 cells compared to the MMC solution and MMC RGD-NPs for 24 or 48 h.

The CCK-8 method was also employed to investigate the proliferation inhibition of MB49 cells by the MMC solution and MMC RGD-NPs. When the MMC concentration was 0.0001~50 μg/mL, the proliferation inhibition of the MB49 cells with NPs was concentration-dependent and, moreover, with the prolongation of the coincubation time, the inhibition ability of the cells was enhanced in a time-dependent manner. On the whole, the inhibition effect of the MMC RGD-NPs was better than that of the MMC solution, as shown in Figure 9. At 24 h, when the MMC concentration is very low, the inhibitory ability of the MMC solution is slightly higher than that of the MMC RGD-NPs, which may be related to the slow release of the MMC RGD NPs in the early stage. However, with the increase in the MMC concentration, the proliferation inhibition ability of the MMC RGD-NPs also increased and was better than that of the MMC solution group. In addition, the IC50 of the MMC solution and the MMC RGD-NPs were calculated. The IC50 corresponding to the 24 h and 48 h administration times of the MMC solution were 5.33 μg/mL and 0.1147 μg/mL, respectively, while the IC50 corresponding to the MMC RGD-NPs group were 1.060 μg/mL and 0.01067 μg/mL, respectively. It can be seen that, compared with the MMC solution, the MMC RGD-NPs have better cell proliferation inhibition ability.

### 3.8. Cellular Uptake of NPs

**Figure 10:**
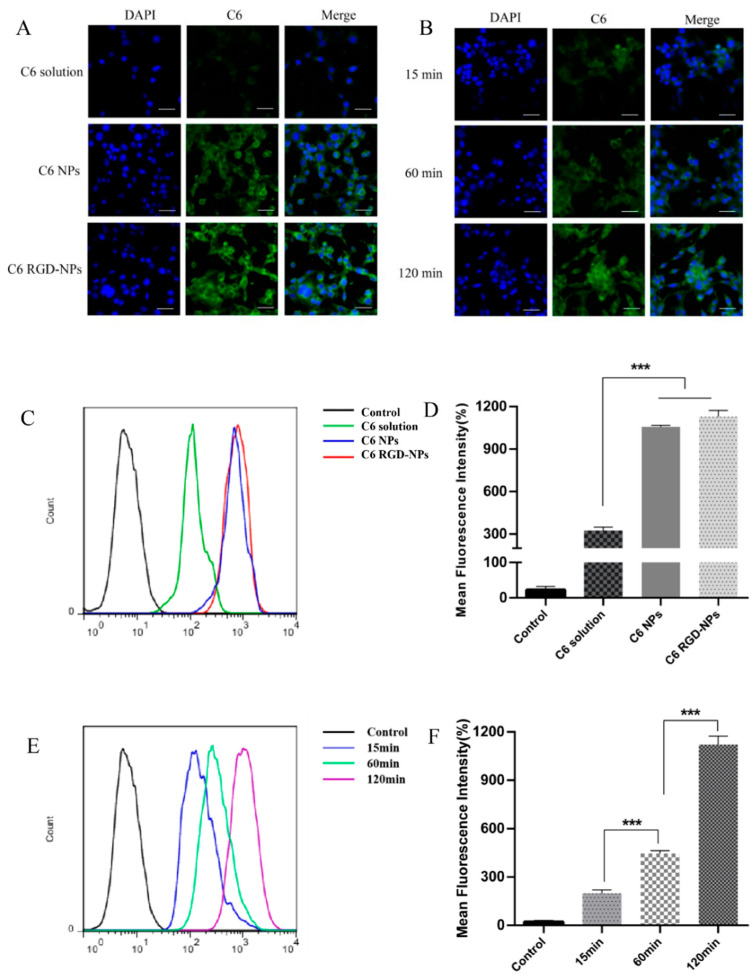
Cell uptake efficiency in vitro. (**A**) Observation of MB49 cells by confocal microscopy after treatment with C6 solution, C6 NPs, and C6 RGD-NPs. Scale bar = 100 µm. (**B**) Observation of MB49 cells by confocal microscopy after treatment with C6 RGD-NPs for 15, 60, and 120 min., respectively. Scale bar = 100 µm. (**C**,**D**) Analysis of the cellular uptake of the control, C6 solution, C6 NPs, and C6 RGD-NPs in MB49 cells by flow cytometry and mean fluorescence intensity. (**E**,**F**) Analysis of the C6 RGD-NP uptake on MB49 cells for 15, 60, and 120 min by flow cytometry and the mean fluorescence intensity, *** *p* < 0.001 to control.

In order to investigate the uptake of NPs by tumor cells, NPs labeled with coumarin 6 (C6) were used and the cellular uptake by MB49 cells was qualitatively investigated by confocal laser scanning microscopy. As shown in Figure 10A, compared with the free C6 solution, obvious fluorescence signals could be observed in the NPs group, and the signals of the C6 RGD-NPs were stronger than that of the C6 NPs. In order to investigate the time dependence of the MB49 cells on the uptake of C6 RGD-NPs, C6 RGD-NPs and MB49 cells were co-incubated for 15, 60, and 120 min, and the laser confocal results were observed, as shown in Figure 10B. A green fluorescence signal appeared after co-incubation for 15 min, indicating that the C6 RGD-NPs had begun to uptake at this time. With the prolongation of time, the fluorescence intensity became stronger and the uptake also increased.

The intracellular uptake efficiency of C6-labeled NPs was also quantitatively determined by flow cytometry. As shown in Figure 10C,D, the intracellular uptake efficiency of the C6 NPs was much stronger than that of free C6, and the C6 RGD-NPs were more efficient compared with the C6 NPs, but there was no significant difference between them. Figure 10E,F show the effect of the different incubation times on C6 RGD-NP uptake. The results show that, with the extension of incubation time, the fluorescence signal intensity increases, indicating an increase in cell uptake.

### 3.9. Cell Apoptosis Assay

**Figure 11:**
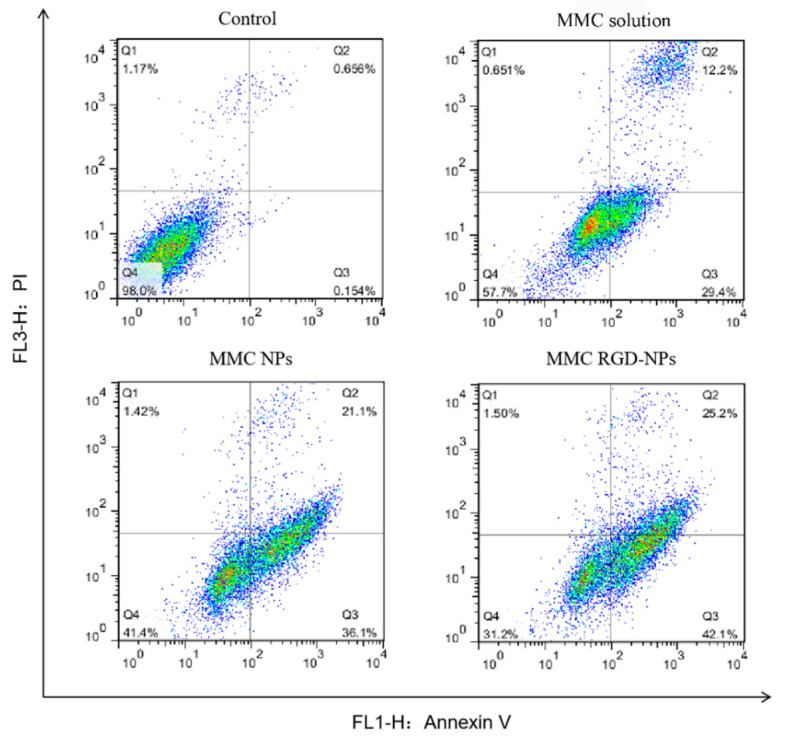
Influence of MMC NPs on cell apoptosis analyzed by flow cytometry.

In order to evaluate the effect of MMC NPs on the apoptosis of tumor cells, MB49 cells were cultured with MMC NPs, MMC RGD-NPs, and MMC solution for 24 h, then the cells were treated with Annexin V-FITC/PI, and the proportion of apoptotic cells was analyzed by flow cytometry. After co-incubation with the tumor cells for 48 h, as shown in Figure 11, the apoptosis rate of the MMC NPs group and the MMC RGD-NPs group was 57.2% and 67.3%, respectively, which was significantly higher than that of the MMC group (41.6%).

### 3.10. Antitumor Evaluation of NPs In Vivo

**Figure 12:**
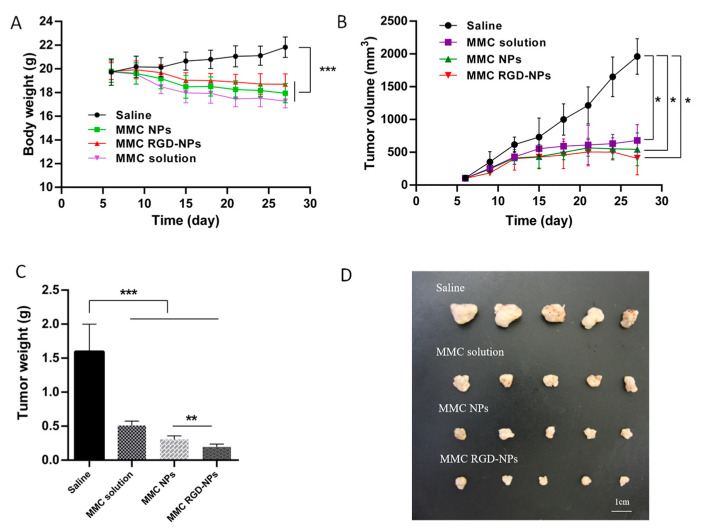
Studies of antitumor efficacy of MMC RGD-NPs in vivo. (**A**) Body weight of mice treated with saline, MMC solution, MMC NPs, MMC RGD-NPs (mean ± SD, *n* = 5). (**B**) Tumor volume of mice treated with saline, MMC solution, MMC NPs, MMC RGD-NPs (mean ± SD, *n* = 5). (**C**) Weight of isolated tumors in groups of saline solution, MMC solution, MMC NPs, MMC RGD-NPs (mean ± SD, *n* = 5). (**D**) Isolated tumor tissues of mice treated with saline, MMC solution, MMC-NPs, MMC-RGD-NPs (mean ± SD, *n* = 5), * *p* < 0.05, ** *p* < 0.01, and *** *p* < 0.001 to control.

MB49-cell tumor-bearing C57BL/6 was selected as an animal tumor model to evaluate the antitumor effect of MMC RGD-NPs in vivo. The tumor-bearing mice, with an average tumor volume of 100 mm^3^, were randomly divided into four groups (saline, MMC solution, MMC NPs, MMC RGD-NPs, *n* = 5). The tumor-bearing mice were administered once every 3 days for 7 times. The tumor volume and body weight of the mice were monitored simultaneously during administration. Changes in the body weight of tumor-bearing mice reflect the toxicity of the drug and carrier, to a certain extent. As shown in Figure 12A, the body weight of mice in the normal saline group increased slowly, which may be related to the noninterference growth of the tumor. The body weight of the mice in the MMC NP and MMC RGD-NP groups showed a trend of slow decrease, while the weight loss in the MMC solution group was more obvious. MMC can also be toxic to normal cells in mice, compared with the MMC solution, and MMC NPs can promote the targeted delivery of MMC to tumor sites, thus reducing the systemic toxicity of MMC.

The changes in the tumor volume and weight of the mice directly reflected the ability of the nanoparticles to inhibit tumor growth. As shown in Figure 12B, the tumor grew rapidly in the saline group. In contrast, the tumors were significantly inhibited when treated with the MMC solution, MMC NPs, and MMC RGD-NPs, especially the latter. Tumors in each group were dissected and weighed three days after the seventh administration. Interestingly, as shown in Figure 12C,D, the average weight of the tumors in the MMC RGD-NPs group was smaller than that in the MMC NPs group (*p* < 0.01), not to mention the MMC solution and saline groups. Then, the tumor inhibition rate was calculated. The tumor inhibition rate of the MMC solution group was 68.0%, and that of the MMC NPs and MMC RGD-NPs groups was 79.9% and 87.8%, respectively.

**Figure 13:**
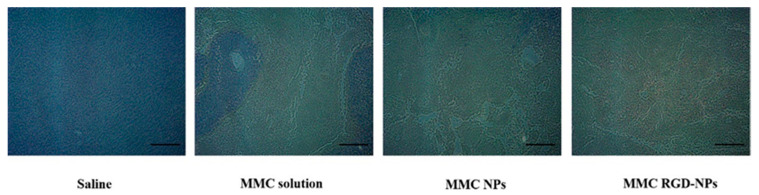
TUNEL assay of tumor tissues isolated from mice treated with saline, MMC solution, MMC NPs, MMC-RGD-NPs, and observed by optical microscope, 100×. Scale bar = 50 μm.

The TUNEL method was used to stain the whole apoptotic nuclei or apoptotic bodies, so as to accurately reflect the biochemical and morphological characteristics of apoptosis [29]. The results of the TUNEL assay (Figure 13) show that almost no apoptotic tumor cells were found in the normal saline group (the brown ones), while a large area of brown apoptotic nuclei was observed in the MMC solution group, the MMC NPs group and the MMC RGD-NPs group, and more apoptotic positive cells and larger apoptotic areas were observed in the MMC NPs and MMC RGD-NPs groups.

**Figure 14:**
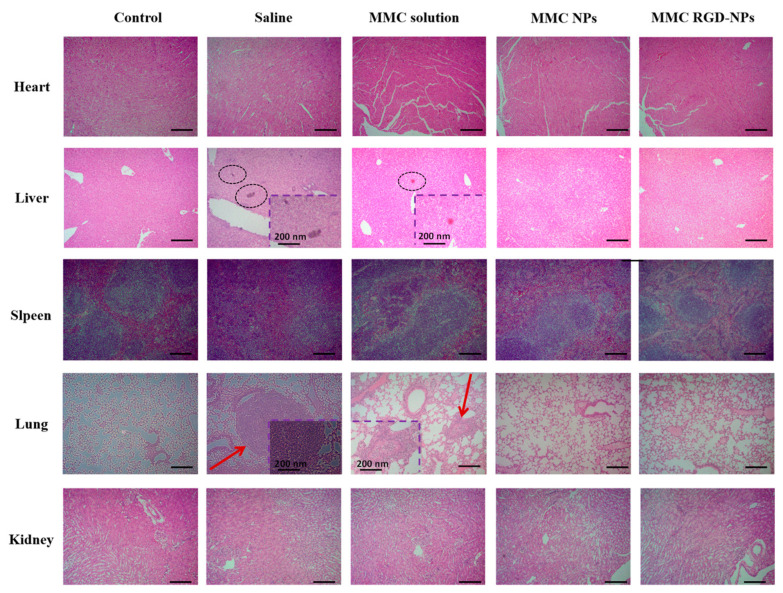
The heart, liver, spleen, lung, and kidney of normal and tumor-bearing mice, treated with saline, MMC solution, MMC NPs, and MMC RGD-NPs, were stained with H&E and observed by optical microscope, 100×. Scale bar = 50 μm. Normal heart, liver, spleen, lung, and kidney were taken as a comparison; circled areas and red arrows show the metastatic areas and the square area was the image of the metastatic areas with large scale.

In order to further observe the metastasis of tumor in various organs, after treatment, the heart, liver, spleen, lung, and kidney tissues of the mice in each group were isolated and stained with H&E, and the results are as shown by Figure 14. In the saline group, there were large tumor metastases and capillary hyperplasia around the metastases. Small tumor metastases were observed in the lung tissues of the mice in the MMC solution group, accompanied by significant alveolar septal thickening and the destruction of the alveolar structure. The structure of the lung tissue in the drug-loaded NPs group was similar to that of the normal mice, and no lung metastasis occurred. According to the H&E staining results of the liver tissues, scattered small nodular tumor liver metastases were found in the liver tissues of mice in the saline and MMC solution groups, while the morphology of the liver tissues in the other groups was normal, and no significant liver metastases were found. According to the H&E staining results of the heart, spleen, and kidney tissues, the morphology of the heart, spleen and kidney tissues in each group was normal, and no tumor metastasis was found. The H&E results suggested that the MMC NPs and MMC RGD-NPs groups showed a better ability to inhibit tumor metastasis than the MMC solution group.

## 4. Discussion

In general, micelles have a good encapsulation ability for lipid-soluble drugs [10]. MMC, as a water-soluble drug, is usually difficult to deliver through micelles, and it is often necessary to modify the structure of the drugs, such as making prodrug or phospholipid complex. Interestingly, the micelles prepared by us can successfully encapsulate the water-soluble MMC. Therefore, we have made an in-depth study and exploration of this experimental phenomenon.

Firstly, molecular docking simulation was conducted and the interaction mode and rules between the drug and polymers were preliminarily discussed. By the molecular docking simulation of MMC and the polymers, the optimal binding conformation can be obtained. Generally, drugs can be trapped inside micelles because of various interactions, such as hydrogen bonding, hydrophobic forces, π–π interactions, van der Waals forces, and so on. It has been widely reported that Fmoc can bind to other substances through π–π stacking interactions, and this feature is often used for the delivery of drugs with π–π bonds, such as paclitaxel [23,30]. Due to the presence of the Fmoc motif in Fmoc-Lys-PEG, π–π stacking interaction may play a significant role in the formation of micelles and drug encapsulation [31]. However, when the molecular docking force between MMC and Fmoc-Lys-PEG was detected by AutoDock Vina, it was found that the hydrogen-bonding interaction was the main force between them, and there was no π–π interaction. The reason for the absence of π–π interaction may be related to the low energy between the π bond and Fmoc in the structure of MMC. Hydrogen bonding is generally thought of as a weak electrostatic attraction that occurs between a hydrogen atom (hydrogen bond donor) and an electronegative atom, such as nitrogen, oxygen, or fluorine (hydrogen bond acceptor) [32], because there are chemical groups, such as the amino and carbonyl groups in the MMC structure and amide bonds in Fmoc-Lys-PEG. Hydrogen bonds can be easily formed between these chemical groups [33]. Through molecular docking simulation, we found the existence of a hydrogen bond force between MMC and Fmoc-Lys-PEG. In order to really verify the hydrogen bond, we further carried out FTIR analysis.

We mainly carried out the FTIR analysis of MMC, blank NPs, and MMC NPs, and proved the existence of a hydrogen bond through the shift of the infrared absorption peak. The results are shown in Figure S3 of the Supporting Information. Since MMC occupies a small proportion in NPs, and the peak of MMC is mostly coincident with Fmoc-Lys-PEG, we cannot judge the peak position change of MMC in the infrared spectrum. Because Fmoc-Lys-PEG forms hydrogen bonding with MMC mainly through the amide bond in the structure [33], we mainly prove the existence of the hydrogen bond by observing the shift of the peak positions of the amide bond in Fmoc-Lys-PEG. The peaks displayed by the amides bond are due to C=O and N–H stretching vibrations, N-H deformations, and mixed vibrations, which are known as “amide bands”, for example the amide I, amide II, amide III [34]. As secondary amides in the Fmoc-Lys-PEG, the N-H stretching vibration absorption is near 3300 cm^−1^, that of the amide I C=O stretching vibration absorption is near 1700 cm^−1^, that of the amide II is near 1500 cm^−1^, and that of the amide III is near 1290 cm^−1^ [34]. As shown in Figure S3, the amides peak of the NPs was 3429.3 cm^−1^ (N–H), 1711.1 cm^−1^ (amide I), 1548.3 cm^−1^ (amide II), and 1250.9 cm^−1^ (amide III), while the amides peak of the MMC NPs was shifted to 3421.3 cm^−1^ (N–H), 1711.1 cm^−1^ (amide I), 1529.4 cm^−1^ (amide II), and 1243.2 cm^−1^ (amide III), which indicates the formation of the H-bond.

From the results of the molecular docking simulation and the infrared spectra, we can draw the conclusion that MMC can be effectively encapsulated in the micelle, and the encapsulation is mainly through hydrogen bonding, while the π–π stacking interaction between Fmoc is beneficial to the formation of the hydrogen-bonding environmental framework in the micelles, thus improving the stability of drug-loaded micelles. Through the investigation of the stability of the carrier at 4 °C, we can see that the as-prepared NPs can remain basically stable within 48 h under the condition of 4 °C. However, with the extension of time, the particle size and PDI of the carrier gradually become larger. As a carrier containing water-soluble drugs, its stability may still have some defects, which will be further explored in future experiments. With regard to the release of the drug, we have firstly considered the in vitro release of MMC NPs in the tumor environment. However, when we tried to search for the physicochemical properties of MMC, we found that it is easily degraded in acidic conditions, even in weaker acids [26,28]. In addition, the antitumor activity of MMC was also related to its structural changes under acidic conditions [27]. Therefore, we only studied the release of MMC NPs under the condition of pH 7.4, and it can be seen from the results that the release process of the micelles was continuous and slow. Therefore, it can be concluded that the as-prepared micelles are stable at 37 °C.

Given that RGD peptides can target integrin receptor that is highly expressed on the endothelial cells of neocapillaries in malignant tumors, RGD was also introduced on the surface of the carrier to further achieve the targeted treatment of bladder cancer. After a series of physical and chemical properties of the NPs were characterized, we also evaluated them in vitro and in vivo. It can be seen from the results that, whether modified with RGD or not, the as-prepared NPs has good biocompatibility and compared with the MMC solution group, the NPs group had a high uptake efficiency, could promote cell apoptosis, and had a strong inhibitory effect on cell proliferation. More importantly, the as-prepared NPs could effectively induce tumor cell apoptosis and inhibit tumor growth and metastasis in vivo. Moreover, according to the tumor growth inhibition effect, the NPs with the RGD target were significantly better than the NPs without the RGD target. These results indicate that MMC-loaded NPs can significantly inhibit tumor growth and that they have an obvious antitumor effect, and that RGD-modified NPs can play an active targeting role, which have better inhibitory efficacy in vivo. However, as shown in the in vitro results, including the tumor cellular uptake and tumor cell apoptosis, although the RGD-modified NPs showed better efficacy, there was no significant difference compared with non-RGD NPs. The reasons may be related to the fact that RGD is more likely to target the endothelial cells of the neocapillaries of bladder cancer in vivo, but the targeting effect on tumor cells in vitro is not obvious. Further experiments will be necessary in our future research.

## 5. Conclusions

In conclusion, in order to solve the problem of the low efficiency and instability of the micelle loading of the water-soluble drug, MMC, we designed and synthesized a new polymer material, Fmoc-Lys-PEG and Fmoc-Lys-PEG-RGD. MMC can be encapsulated into micelles by hydrogen-bonding interactions. By the molecular docking simulation of MMC and the material, the optimal binding conformation can be obtained, and it is calculated that the main interaction force between them is hydrogen bonding. As MMC is a first-line treatment for bladder cancer, we further evaluated the MMC NPs antitumor efficiency for bladder cancer in vivo and in vitro. The results show that the MMC NPs had a high uptake efficiency, could promote cell apoptosis, and had a strong inhibitory effect on cell proliferation. Moreover, MMC NPs can effectively induce tumor cell apoptosis and inhibit tumor growth and metastasis in vivo, and RGD-modified NPs exhibit a better tumor growth inhibition effect. Therefore, MMC NPs are promising drug carriers that have broad potential for the delivery of MMC drugs and the treatment of bladder cancer.

## Figures and Tables

**Table 1:** Screening results of hydration media.

Types	Water Dissolution Solvent	Particle Size (nm)	PDI
MMC NPs	Water	174.8 ± 7.0	0.297 ± 0.062
	0.9% NaCl	160.2 ± 4.3	0.267 ± 0.035
	PBS	115.5 ± 6.4	0.219 ± 0.027
MMC RGD-NPs	Water	155.4 ± 7.2	0.283 ± 0.045
	0.9% NaCl	146.2 ± 4.4	0.164 ± 0.041
	PBS	107.6 ± 2.2	0.137 ± 0.013

**Table 2:** The screening results of the drug and carrier molar ratios.

Types	Carrier:Drug ^1^	Size (nm)	PDI
MMC NPs	—	119.9 ± 9.0	0.275 ± 0.018
	0.5:1	141.1 ± 4.7	0.271 ± 0.028
	1:1	134.1 ± 2.8	0.277 ± 0.018
	2.5:1	115.5 ± 6.4	0.219 ± 0.027
MMC RGD-NPs	—	107.8 ± 6.7	0.398 ± 0.059
	0.5:1	134.7 ± 0.9	0.259 ± 0.026
	1:1	123.6 ± 1.7	0.214 ± 0.029
	2.5:1	107.6 ± 2.2	0.137 ± 0.013

^1^ Molar ratio.

**Table 3:** Hemolysis of MMC-NPs and MMC RGD-NPs.

Concentration (mg/mL)		0.10	0.25	0.50	0.75	1.0
**Degree of hemolysis (%)**	MMC NPs	0.08	0.29	0.50	1.19	1.84
	MMC RGD-NPs	0.10	0.13	0.74	1.32	2.13

## Data Availability

Data is contained within the article.

## Supplementary Materials

The following are available online at https://www.mdpi.com/article/10.3390/pharmaceutics13111776/s1, Figure S1: Size distribution of MMC NPs (A) and MMC RGD-NPs (B), Figure S2: Zeta potential distribution of MMC NPs (A) and MMC RGD-NPs (B), Figure S3: Fourier-transform infrared spectrum of Blank NPs, MMC NPs and MMC.
