# Correction: Qi et al. Development of Mitomycin C-Loaded Nanoparticles Prepared Using the Micellar Assembly Driven by the Combined Effect of Hydrogen Bonding and π–π Stacking and Its Therapeutic Application in Bladder Cancer. *Pharmaceutics* 2021, *13*, 1776

**DOI:** 10.3390/pharmaceutics17060733

**Published:** 2025-06-03

**Authors:** Lingling Qi, Chao Liu, Yingying Zhang, Zheao Zhang, Hongxia Duan, Heming Zhao, Xin Xin, Liqing Chen, Mingji Jin, Youyan Guan, Zhonggao Gao, Wei Huang

**Affiliations:** 1 State Key Laboratory of Bioactive Substance and Function of Natural Medicines, Department of Pharmaceutics, Institute of Materia Medica, Chinese Academy of Medical Sciences and Peking Union Medical College, Beijing 100050, China; 2 Department of Urology, National Cancer Center/National Clinical Research Center for Cancer/Cancer Hospital, Chinese Academy of Medical Sciences and Peking Union Medical College, Beijing 100021, China

## Error in Figure

In the original publication [1], there was a mistake in Figure 10 as published. Figure 10A,B were unintentionally duplicated. The corrected Figure 10 appears below. The authors state that the scientific conclusions are unaffected. This correction was approved by the Academic Editor. The original publication has also been updated.

## Figures and Tables

**Figure 10:**
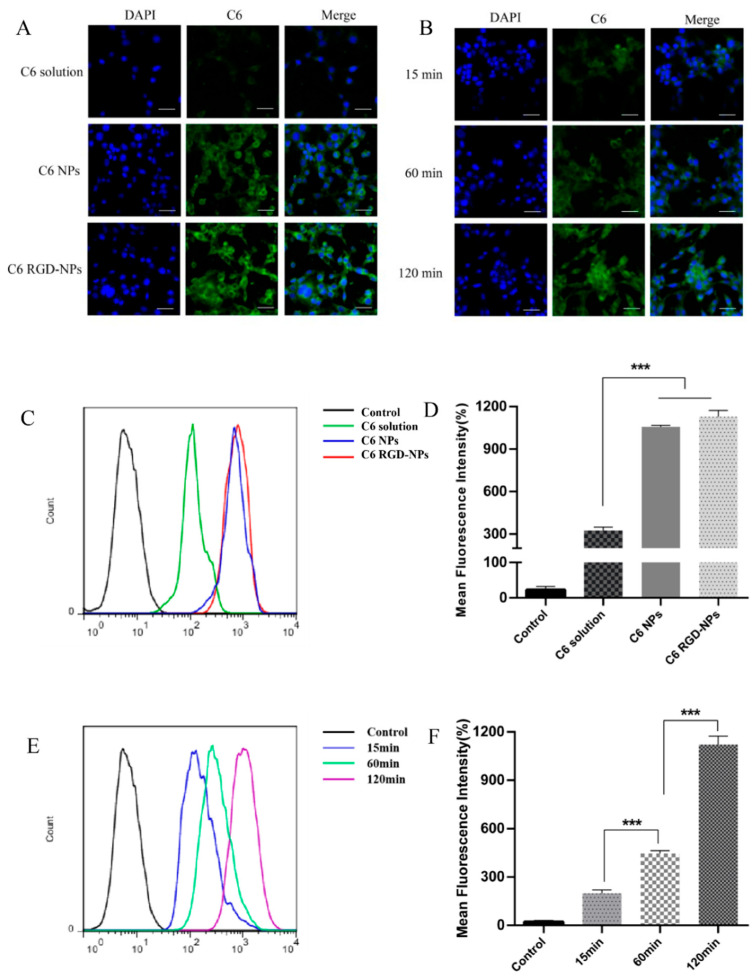
Cell uptake efficiency in vitro. (**A**) Observation of MB49 cells by confocal microscopy after treatment with C6 solution, C6 NPs, and C6 RGD-NPs. Scale bar = 100 µm. (**B**) Observation of MB49 cells by confocal microscopy after treatment with C6 RGD-NPs for 15, 60, and 120 min., respectively. Scale bar = 100 µm. (**C**,**D**) Analysis of the cellular uptake of the control, C6 solution, C6 NPs, and C6 RGD-NPs in MB49 cells by flow cytometry and mean fluorescence intensity. (**E**,**F**) Analysis of the C6 RGD-NP uptake on MB49 cells for 15, 60, and 120 min by flow cytometry and the mean fluorescence intensity, *** *p* < 0.001 to control.
