# Supplementary material for: Development of Mitomycin C-Loaded Nanoparticles Prepared Using the Micellar Assembly Driven by the Combined Effect of Hydrogen Bonding and π–π Stacking and Its Therapeutic Application in Bladder Cancer
Source: Pharmaceutics. 2021 Oct 25;13(11):1776. doi: 10.3390/pharmaceutics13111776 (PMC8624376; doi:10.3390/pharmaceutics13111776)
Supplement: Supplementary file 1 [file pharmaceutics-13-01776-s001.zip › pharmaceutics-1369916-supplementary.pdf]

pharmaceutics

Supplementary Materials: Development of Mitomycin C-Loaded Nanoparticles Prepared Using the Micellar Assembly Driven by the Combined Effect of Hydrogen Bonding and π–π Stacking and Its Therapeutic Application in Bladder Cancer

Lingling Qi, Chao Liu, Yingying Zhang, Zheao Zhang, Hongxia Duan, Heming Zhao, Xin Xin, Liqing Chen, Mingji Jin, Jingzhe Jin, Youyan Guan, Zhonggao Gao, Wei Huang

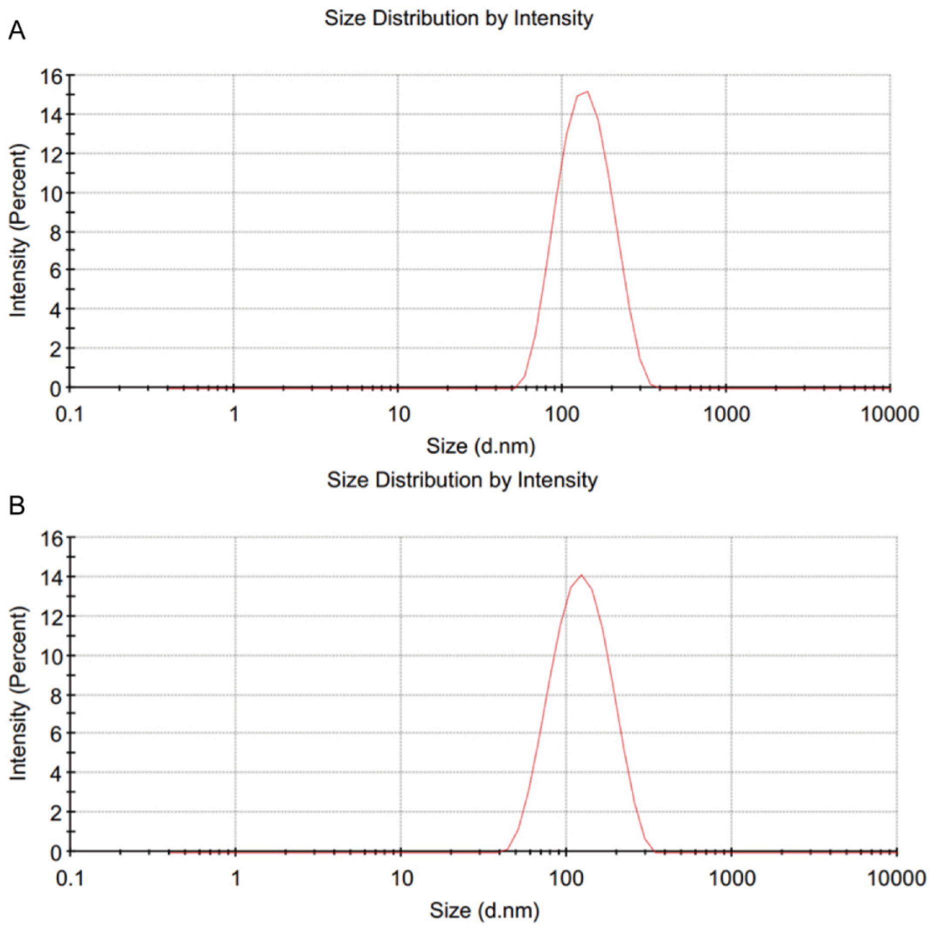

Figure S1. Size distribution of MMC NPs (**A**) and Size distribution of MMC RGD-NPs (**B**).

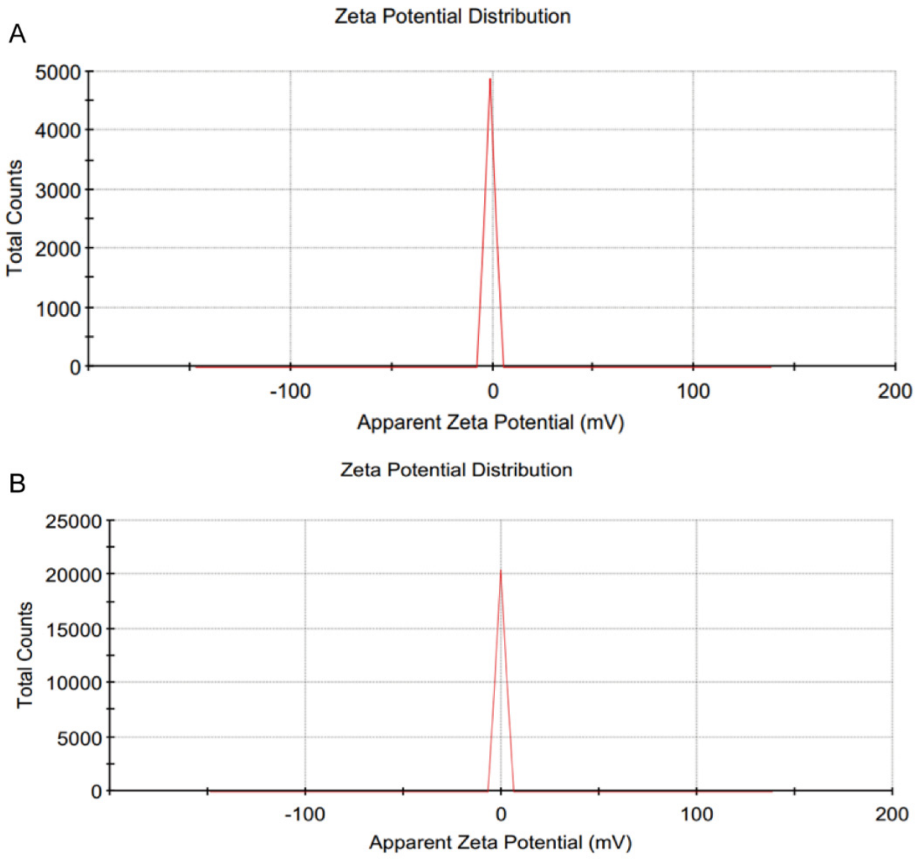

Figure S2. Zeta Potential Distribution of MMC NPs (**A**) and Zeta Potential Distribution of MMC RGD-NPs (**B**).

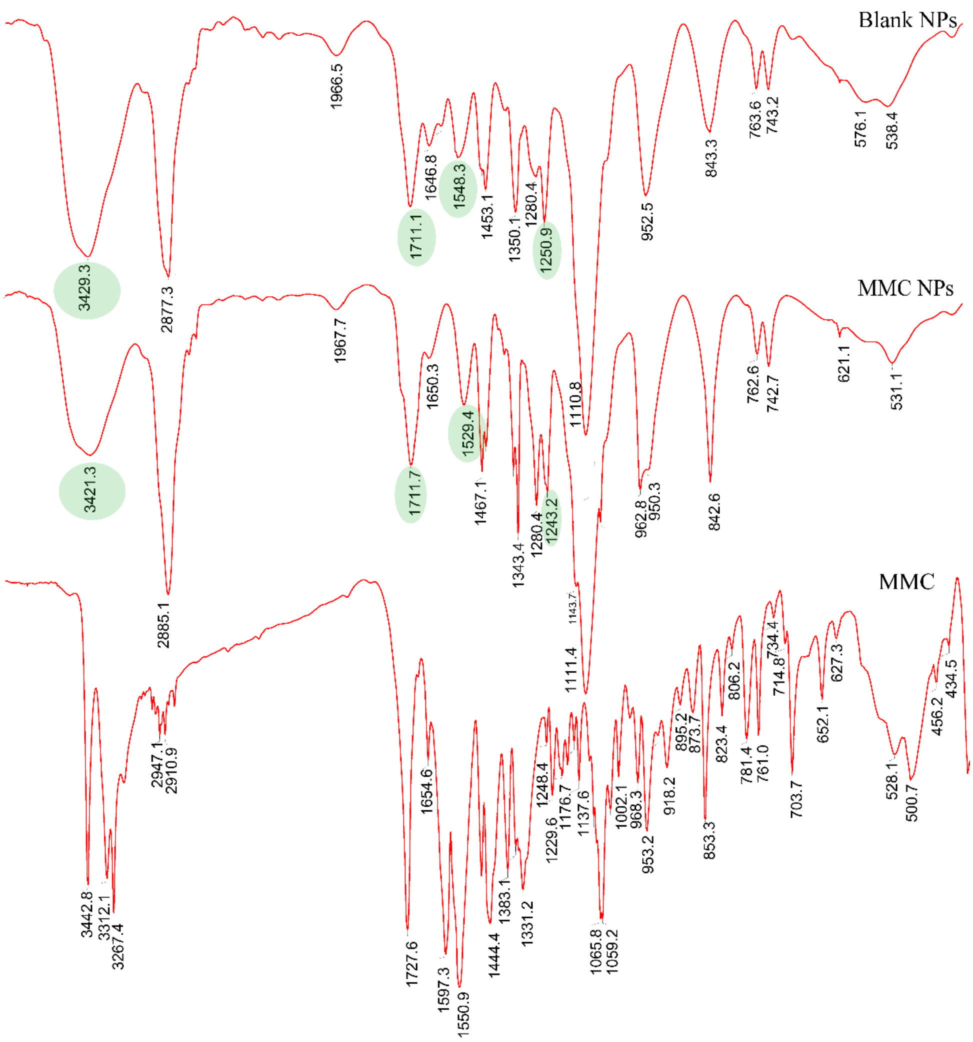

Figure S3. Fourier-transform infrared spectrum (FTIR) of Blank NPs, MMC NPs and MMC.
